# Scale of Medical Fees for the Kingdom of Bavaria

**Published:** 1837-01

**Authors:** 


### SCALE OF MEDICAL FEES FOR THE KINGDOM OF BAVARIA, FIXED BY A ROYAL ORDONNANCE, BEARING DATE MARCH 31, 1836.

### I. *Fees for Physicians and Surgeons, regularly educated and licenced.*

A. For Visits, Consultations, Cadaveric Examinations, Certificates, Chemical Examinations.

| | From Fl. Kr.* | to Fl. Kr. |
|---|---|---|
| 1. For visits to patients residing in the same place with the doctor, the suburbs included: | | |
| *a.* For the first visit | 0 30 | to 1 12 |

* The English equivalent for a floren is about twenty pence (1s. 8*d*.), and for a kreutzer about one-third of a penny, there being sixty kreutzers in the floren.

| | From Fl. | Kr. | to Fl. | Kr. |
|---|---|---|---|---|
| *b.* For every other visit . . . | 0 | 15 | 0 | 45 |
| *c.* For night visits, (i. e. from 9 P.M. to 6 A.M.) | the above doubled. | | | |
| 2. For prescribing at the patient's home, with or without a written prescription . . . | 0 | 12 | 0 | 36 |
| If there happen to be in a family, or in a public establishment, several members sick at the same time, the fee is increased by one half. | | | | |
| 3. For consultations with one or more physicians: | | | | |
| *a.* For the first (by day) . . . | 1 | 30 | 5 | 0 |
| (by night) . . . | one half more. | | | |
| *b.* For the other ordinary visits (by day) . | 0 | 24 | 1 | 12 |
| (by night) . | the above doubled. | | | |
| 4. For advice by correspondence: | | | | |
| *a.* With a patient . . . . | 1 | 0 | 3 | 0 |
| *b.* With a doctor . . . . | 2 | 0 | 4 | 0 |
| *c.* With a written case, prescriptions, and opinion | 3 | 0 | 9 | 0 |
| 5. For the required or necessary stay of the physician with a patient: | | | | |
| *a.* By day, for each hour . . . | 0 | 48 | 1 | 30 |
| *b.* By night, ditto . . . . | 1 | 0 | 2 | 0 |
| 6. Certificates: | | | | |
| *a.* For a patient previously known, (exclusive of the stamp) . . . . | 0 | 36 | 1 | 12 |
| *b.* For an unknown patient, after enquiry into his state of health, (excl. of the stamp) . | 1 | 12 | 2 | 24 |
| 7. For a Report to some public body: | | | | |
| *a.* Simple . . . . . . | 0 | 48 | 1 | 24 |
| *b.* With a history of the case, or stated opinion | 3 | 0 | 8 | 0 |
| 8. For the inspection of a corpse, such as is requisite in a judicial case, with or without a report: | | | | |
| *a.* Previously to the supervention of putrefaction | 1 | 30 | 3 | 0 |
| *b.* After ditto . . . . . | 2 | 0 | 4 | 0 |
| 9. For conducting a cadaveric examination, with the minuteness requisite in criminal cases, with or without a report: | | | | |
| *a.* Previously to putrefaction - . | 3 | 0 | 5 | 0 |
| *b.* After ditto . . . . . | 4 | 0 | 8 | 0 |
| *c.* For a child . . . . . | 2 | 0 | 4 | 0 |
| 10. For attending at an examination of a dead body: | | | | |
| *a.* Before putrefaction . . . . | 2 | 0 | 3 | 0 |
| *b.* After ditto . . . . . | 3 | 0 | 5 | 0 |
| 11. For embalming a body: | | | | |
| *a.* Of a child, (the materials not included) . | 15 | 0 | 30 | 0 |
| *b.* Of an adult, (ditto) . . | 30 | 0 | 60 | 0 |
| 12. For the Report of a post-mortem examination, (according to the difficulty and importance of the case) | 3 | 0 | 6 | 0 |
| 13. For being present at the inspection of the body of an animal suspected of rabies or other infectious disorder . . . . . . | 1 | 30 | 2 | 30 |
| Compensation must be made over and above for loss in clothes and instruments. | | | | |
| 14. Examination of a druggist's shop, on the requisition of a complainant . . . . | 10 | 0 | 15 | 0 |
| 15. Chemical examination after poisoning, with a report | 6 | 0 | 24 | 0 |
| 16. requiring various tests, as, e.g. of vinegar . . | 2 | 30 | 4 | 0 |
| 17. of wine, beer, &c. . . | 2 | 0 | 4 | 0 |

| | From Fl. | Kr. | to Fl. | Kr. |
|---|---|---|---|---|
| N.B. In these examinations, the price of tests may be also reckoned over and above. The fees for No. 15, 16, and 17, the physician only receives when he undertakes the examination personally; for the mere overlooking this, he only receives one half. | | | | |
| 18. If any of the foregoing categories requires the physician to travel from his usual place of residence, he charges, over and above, for the time consumed, his maintenance inclusive: | | | | |
| *a.* For half an hour | 0 | 30 | 0 | 48 |
| *b.* For the first hour | 1 | 0 | 1 | 36 |
| *c.* For each of the three following hours | 0 | 30 | 0 | 48 |
| Besides a decent conveyance; or, in lieu of this, a sum equivalent to the ordinary carriage-hire. | | | | |
| 19. If the absence exceeds four hours, the fee is 5 fl.; and if the distance renders it necessary to be from home a night, it is 8 fl. | | | | |

B. For Chirurgical Operations.

| | From Fl. | Kr. | to Fl. | Kr. |
|---|---|---|---|---|
| 20. For slight, easy, and short operations, and with the simplest and most common instruments; e.g. simple incisions, the taxis, punctures, sutures, ligature of vessels, removal of foreign bodies from cavities easily accessible, introduction of the catheter, extirpation of small tumours, and the like | 1 | 0 | 10 | 0 |
| N.B. In the application of this regulation, regard is to be had to the difference of the operation; advancing from the simple incision up to the difficult scientific operations. | | | | |
| 21. For the greater operations, consisting of several parts, and requiring a special apparatus of instruments, as well as a frequently repeated and close practice; e.g. trepanning the skull, the hare-lip operation, amputation of the female mamma, of the larger limbs, operation for hydrocele, fistula in ano, castration, setting of dislocated and fractured limbs, removal of large tumours, &c. | 10 | 0 | 30 | 0 |
| 22. For the most capital operations, such as require for their performance the greatest skill; e.g. the operation for cataract, for artificial pupil, the rhinoplastic operation, bronchotomy, operation for hernia, lithotomy, for aneurism, &c. | 30 | 0 | 80 | 0 |

C. For Obstetrical Operations.

| | From Fl. | Kr. | to Fl. | Kr. |
|---|---|---|---|---|
| 23. For examination to ascertain the existence of pregnancy, of previous delivery, diseases of the female organs, &c. | | | | |
| *a.* Of a sound person | 0 | 48 | 1 | 20 |
| *b.* Of an infected person | 1 | 12 | 2 | 40 |
| 24. For an easy, natural labour | 5 | 0 | 11 | 0 |
| 25. For a natural labour, lasting one day and night | 8 | 0 | 15 | 0 |
| 26. For twins, one half more. | | | | |
| 27. For completing a breech or foot presentation, without previous turning, and without the use of forceps | 5 | 0 | 12 | 0 |
| 28. For delivery requiring turning: | | | | |
| *a.* In ordinary cases | 5 | 0 | 12 | 0 |
| *b.* In cases rendered difficult from the position of the child, or strong contraction of the uterus | 10 | 0 | 20 | 0 |

| | From Fl. | Kr. | to | Fl. | Kr. |
|---|---|---|---|---|---|
| 29. For a delivery by the forceps: | | | | | |
| *a.* For an easy case | 5 | 0 | .. | 12 | 0 |
| *b.* For a difficult case, from the high position or the wedging of the head | 8 | 0 | .. | 16 | 0 |
| 30. For a forceps delivery, with perforation | 12 | 0 | .. | 20 | 0 |
| 31. For turning, complicated with dismemberment | 12 | 0 | .. | 20 | 0 |
| 32. For removing a strongly encysted or adherent placenta | 5 | 0 | .. | 10 | 0 |
| 33. For the operation of imperforate vagina | 4 | 0 | .. | 8 | 0 |
| 34. For laying open a cicatrised os uteri | 6 | 0 | .. | 12 | 0 |
| 35. For the Cæsarean operation, without regard to whether the child live or die: | | | | | |
| *a.* On a living person | 15 | 0 | .. | 30 | 0 |
| *b.* On a dead person | 6 | 0 | .. | 12 | 0 |
| 36. Extirpation of the uterus | 24 | 0 | .. | 40 | 0 |
| 37. For extirpation of part of the uterus | 12 | 0 | .. | 30 | 0 |
| 38. For replacing an inverted uterus | 6 | 0 | .. | 12 | 0 |
| 39. For replacing a descent of the vagina, uterus, or rectum | 2 | 0 | .. | 4 | 0 |
| 40. For applying a pessary | 1 | 30 | .. | 3 | 0 |
| 41. For inducing an artificial miscarriage | 6 | 0 | .. | 12 | 0 |
| 42. For manual assistance in threatened uterine hemorrhage | 2 | 0 | .. | 8 | 0 |
| 43. For the ligature of a vaginal, uterine, or anal polypus | 4 | 0 | .. | 8 | 0 |
| 44. For removal of one of the nymphæ | 1 | 0 | .. | 2 | 0 |
| 45. For removal of an immature ovum or mole | 2 | 0 | .. | 4 | 0 |
| 46. For injections | 0 | 48 | .. | 1 | 20 |

II. *Fees for Dentists.*

| | | | | | |
|---|---|---|---|---|---|
| 47. For the drawing of a tooth | 0 | 12 | .. | 0 | 48 |
| 48. For the drawing of a root or stump | 0 | 24 | .. | 1 | 0 |
| 49. For cauterizing a tooth, with the actual cautery | | | | | |
| 50. For filling up a tooth | 0 | 12 | .. | 0 | 48 |
| 51. For cleaning the whole set of teeth | 2 | 0 | .. | 4 | 0 |
| 52. For boring or piercing a tooth | 0 | 30 | .. | 1 | 0 |
| 53. For filing a stump or filing through a tooth | 0 | 30 | .. | 1 | 0 |
| 54. For the operation of a tooth-fistula (?) | 1 | 12 | .. | 2 | 0 |
| 55. For straightening an irregular tooth in a child | 0 | 36 | .. | 1 | 12 |
| 56. For preparing and fixing an artificial tooth, the previous operation included | 2 | 0 | .. | 5 | 0 |
| 57. For fastening a tooth | 0 | 30 | .. | 0 | 48 |
| 58. For scarifying the gums, or for any other slight operation on the gums | 0 | 12 | .. | 0 | 36 |

N.B. 1. If any of the foregoing operations is performed out of the dentist's own house, an additional charge of 20 krs. is made.

2. When several teeth require the same operation, then half the stated charge is to be made for each after the first.

3. The materials expended in certain operations,—as, for example, gold,—is to be charged separately.

III. *For Cuppers and Apothecaries.*

| | | | | | |
|---|---|---|---|---|---|
| 59. For the application of a dry cupping-glass | 0 | 2 | .. | 0 | 4 |
| For each succeeding one, one-half ditto. | | | | | |
| 60. For the application of the scarificator | 0 | 6 | .. | 0 | 12 |
| 61. For bloodletting in the arm or foot | 0 | 12 | .. | 0 | 24 |
| 62. For the application of leeches, (exclusive of their price,) for each leech | 0 | 3 | .. | 0 | 6 |
| 63. For scarification with the knife | 0 | 12 | .. | 0 | 24 |
| 64. For the application of a blister or sinapism | 0 | 6 | .. | 0 | 24 |
| 65. For the application of an exutory | 0 | 12 | .. | 0 | 24 |

| | | From Fl. | Kr. | to | Fl. | Kr. |
|---|---|---|---|---|---|---|
| 66 | For making an issue . . . . . | 0 | 20 | .. | 0 | 30 |
| 67. | For introducing a seton . . . . | 0 | 36 | .. | 1 | 0 |
| 68. | For giving an enema . . . . | 0 | 12 | .. | 0 | 24 |
| 69. | of tobacco smoke . . | 0 | 24 | .. | 0 | 40 |
| 70. | For injecting a fistulous opening . . . | 0 | 12 | .. | 0 | 24 |
| 71. | For opening an abscess . . . . | 0 | 15 | .. | 0 | 30 |
| 72. | For extirpating a corn, wart, or small tumour . | 0 | 30 | .. | 1 | 0 |
| | For several, half the price for each. | | | | | |
| 73. | For stopping bleeding at the nose, without instruments | 0 | 24 | .. | 0 | 36 |
| | with instruments . | 1 | 0 | .. | 2 | 0 |
| 74. | For drawing off the urine: | | | | | |
| | *a.* In men . . . . . . | 0 | 30 | .. | 1 | 0 |
| | *b.* In women . . . . . . | 0 | 15 | .. | 0 | 24 |
| | When repeated, half the above. | | | | | |
| 75. | For the application of caustic . . . | 0 | 12 | .. | 0 | 24 |
| 76. | For the first dressing of a simple wound, including the visit | 0 | 20 | .. | 1 | 0 |
| 77. | For the first dressing of a complicated wound with fracture, gangrene, &c., the visit included . . | 0 | 30 | .. | 1 | 12 |
| 78. | For each succeeding visit in the above cases: | | | | | |
| | *a.* By day . . . . . . | 0 | 9 | .. | 0 | 12 |
| | *b.* By night . . . . . . | 0 | 15 | .. | 0 | 24 |
| | *c.* In the country, by the hour, going and coming | 0 | 15 | .. | 0 | 24 |

IV. *For Midwives.*

| | | Fl. | Kr. | to | Fl. | Kr. |
|---|---|---|---|---|---|---|
| 79. | For examination of a pregnant or other woman . | 0 | 24 | .. | 0 | 36 |
| 80. | For a common delivery, not lasting over twelve hours | 1 | 0 | .. | 3 | 0 |
| 81. | For every hour after twelve . . . . . | 0 | 6 | .. | 0 | 12 |
| 82. | For twins, double the above. | | | | | |
| 83. | For a delivery, with turning . . . . | 1 | 30 | .. | 2 | 0 |
| 84. | For removing an imperfect ovum or mole . . | 0 | 48 | .. | 1 | 30 |
| 85. | For giving an enema or vaginal injection, not during pregnancy or the puerperal state . . . | 0 | 9 | .. | 0 | 15 |
| 86. | For the introduction of the catheter . . . | 0 | 12 | .. | 0 | 20 |
| 87. | For resuscitating an infant apparently dead, without perceptible pulsation of the heart or respiration . | 1 | 30 | .. | 3 | 0 |
| 88. | For assisting at a birth superintended by an accoucheur | 1 | 0 | .. | 2 | 0 |
| 89. | For each visit to a lying-in woman, including the usual care of the mother and child, when the distance out and home does not exceed a league . . . | 0 | 12 | .. | 0 | 18 |

N.B. 1. For loss of time in the execution of their office beyond the precincts of their place of residence, midwives, like cuppers, charge half the fee appropriated to surgeons, viz. for each hour 9 to 15 kreutzers.

2. For medicaments supplied by the midwives, one-half more may be charged than by the druggists.

3. For examinations and attendances in cases of contagious or disgusting diseases, one-half more than the customary fee may be charged.

V. *For Nurses.*

| | | Fl. | Kr. | to | Fl. | Kr. |
|---|---|---|---|---|---|---|
| 90. | For ordinary, not contagious diseases, for every twenty-four hours, (including food and drink thrice a day) | 0 | 24 | .. | 0 | 40 |
| 91. | When food and drink are not supplied, the allowance for these will be regulated by the customs of the place. | | | | | |
| 92. | In cases of contagious or very disgusting diseases, and in madness, the charge is one-half more. | | | | | |
| 93. | If the nurse is called into the country, for every league the same allowance as for midwives (including food) . | 0 | 9 | .. | 0 | 15 |

VI. *For Veterinary Surgeons.*

94. Veterinary surgeons, in relation to their duties in the case of epidemic diseases or other state duties, are to be paid as country doctors and surgeons of the first class.

N.B. Owing to the recent establishment of this department of medicine, as the customary charges, from which a scale might be framed, have not yet been fixed, the fixing a fee for individual cases of service is for the present postponed. *Henke's Zeitschrift für die Staatsarzneikunde*, iii. heft, 1836.

N.B. In the present statistical relations of the medical profession in England, the preceding Tables of Fees cannot fail to be interesting to our readers. It is worthy of notice, that the one comes from the freest and the other from one of the most despotic states; and that in the former the scale was fixed by the practitioners themselves, in the latter by the government. Even in Bavaria, however, doubtless the scale of fees originated with the profession.—Eds.

---

